# The cost‐effectiveness of pharmacotherapy and lifestyle intervention in the treatment of obesity

**DOI:** 10.1002/osp4.390

**Published:** 2019-12-10

**Authors:** Minyi Lee, Brianna N. Lauren, Tiannan Zhan, Jin Choi, Matthew Klebanoff, Barham Abu Dayyeh, Elsie M. Taveras, Kathleen Corey, Lee Kaplan, Chin Hur

**Affiliations:** ^1^ Gastroenterology Division Massachusetts General Hospital Boston Massachusetts; ^2^ Institute for Technology Assessment Massachusetts General Hospital Boston Massachusetts; ^3^ Department of General Medicine Columbia University Irving Medical Center New York New York; ^4^ Healthcare Innovation Research and Evaluation Columbia University Irving Medical Center New York New York; ^5^ Yale School of Medicine New Haven Connecticut; ^6^ Division of Gastroenterology and Hepatology Mayo Clinic Rochester Minnesota; ^7^ Department of Pediatrics Massachusetts General Hospital Boston Massachusetts; ^8^ Harvard Medical School Boston Massachusetts

**Keywords:** cost‐effectiveness analysis, obesity, pharmacotherapy, weight loss

## Abstract

**Background:**

The Food and Drug Administration has approved several pharmacotherapies for the treatment of obesity. This study assesses the cost‐effectiveness of six pharmacotherapies and lifestyle intervention for people with mild obesity (body mass indices [BMIs] 30 to 35).

**Methods:**

A microsimulation model was constructed to compare seven weight loss strategies plus no treatment: intensive lifestyle intervention, orlistat, phentermine, phentermine/topiramate, lorcaserin, liraglutide, and semaglutide. Weight loss, quality‐of‐life scores, and costs were estimated using clinical trials and other published literature. Endpoints included costs, quality‐adjusted life years (QALYs), and incremental cost‐effectiveness ratios (ICERs) with a willingness‐to‐pay (WTP) threshold of $100 000/QALY. Results were analysed at 1‐, 3‐, and 5‐year time horizons.

**Results:**

At each of the three follow‐up periods, phentermine was the cost‐effective strategy, with ICERs of $46 258/QALY, $20 157/QALY, and $17 880/QALY after 1, 3, and 5 years, respectively. Semaglutide was the most effective strategy in the 3‐ and 5‐year time horizons, with total QALYs of 2.224 and 3.711, respectively. However, the ICERs were prohibitively high at $1 437 340/QALY after 3 years and $576 931/QALY after 5 years. Deterministic and probabilistic sensitivity analyses indicated these results were robust.

**Conclusions:**

Phentermine is the cost‐effective pharmacologic weight‐loss strategy. Although semaglutide is the most effective, it is not cost‐effective because of its high price.

## 1 INTRODUCTION

An estimated 70% of the population in the United States have overweight or obesity, a threefold increase over the last 40 years.^1^ Worldwide, an estimated 650 million adults live with overweight or obesity.^2^ With the large population of affected individuals, the economic costs of obesity are substantial. The cost of medical care related to obesity in adults is estimated at $85.7 billion in the United States^3^ but could be as much as $209.7 billion.^4^ On a global level, obesity‐related complications are estimated to cost $1.2 trillion by 2025, almost half of which will be spent in the United States alone.^5^ Weight reduction of as little as 5% in individuals with obesity is associated with improved health outcomes and reduced incidence of obesity‐related comorbidities, including cardiovascular disease and diabetes.^6^ Effective treatments and interventions are crucial but remain elusive.

Lifestyle intervention, with diet, physical activity, and behaviour modification, is the standard first‐line therapy for overweight and obesity. However, adaptive physiologic responses, such as increased appetite and decreased resting metabolic rate, make it difficult to maintain weight loss through lifestyle intervention alone.^7^ After initial weight loss in the first year, weight regain occurs at an average rate of 1 to 2 kg y^−1^.^6^, ^8^ For this reason, individuals with a body mass index (BMI) of at least 30 kg m^−2^ or a BMI of at least 27 kg m^−2^ with weight‐related comorbidities are eligible for pharmacotherapy.^7^, ^9^

The Food and Drug Administration (FDA) has approved several pharmacotherapies to treat overweight and obesity. As more agents enter the market, comparative efficacy and economic burdens are difficult to discern. Randomized, placebo‐controlled trials for orlistat, lorcaserin, liraglutide, and phentermine/topiramate have shown promising results for individuals with obesity, leading to FDA approval. In each trial, the majority of participants lost 5% to 10% of their baseline weight within the first year.^10^, ^11^, ^12^, ^13^

Phentermine and semaglutide are not FDA approved for long‐term treatment of obesity. However, both medications demonstrate efficacy and increasing use within the medical community.^5^, ^14^, ^15^ Phentermine is the most commonly prescribed pharmacotherapy for weight‐loss in the United States, despite FDA approval for only short‐term use (90 days) and a lack of large, long‐term clinical trials.^15^, ^16^, ^17^ In a randomized, placebo‐controlled trial, individuals with overweight or obesity lost an average of 9.3% of baseline body weight after 14 weeks.^18^ Once‐weekly semaglutide is FDA approved only as treatment for type 2 diabetes. However, a recent phase 2, randomized, double‐blind, placebo and active controlled trial with nondiabetic individuals found promising results for daily semaglutide as obesity treatment (n = 957). Results indicated that semaglutide doses of 0.2 mg d^−1^ or more significantly increased weight loss compared with both liraglutide (3.0 mg d^−1^) and placebo.^5^ In addition, a recent randomized, placebo‐controlled trial demonstrated that diabetic patients on semaglutide can sustain weight loss for at least 2 years.^19^

A clinical trial comparing all available pharmacotherapies for obesity is unlikely to occur in the future for various reasons, including the high costs and large sample sizes that would be required. The purpose of this study is to compare and analyse the cost‐effectiveness of six pharmacotherapies and intensive lifestyle intervention in patients with mild obesity (BMI between 30 and 35). It builds on previous cost‐effectiveness analyses^20^, ^21^ by focusing exclusively on pharmacotherapy treatment for a specific population and including phentermine and semaglutide. In addition, this analysis follows patients on long‐term treatment (over 1 year), in accordance with the increasing recognition of obesity as a chronic, relapsing medical disease.^22^

## 2 METHODS

### 2.1 Model overview

A microsimulation model was developed using Python 3.6.5 to assess the cost‐effectiveness of seven strategies plus no treatment: intensive lifestyle intervention (ILI), phentermine/topiramate (7.5/46 mg daily), liraglutide (3.0 mg daily), semaglutide (0.4 mg daily), orlistat (120 mg TID), lorcaserin (10 mg BID), and phentermine (37.5 mg daily). In addition, a scenario analysis that excludes phentermine was performed since phentermine is not FDA approved for long‐term treatment. Liraglutide and semaglutide are subcutaneous injections, while all other pharmacotherapies are oral. For the base case, 100 000 patients were modelled, with 75% females and initial age of 40 based on patient populations in the clinical trials. Initial BMI was 32.5 kg m^−2^, representing the midpoint BMI within the mildly obese classification (BMIs 30 to 35 kg m^−2^). Initial quality of life (QOL) was 0.720, based on estimates for people with mild obesity in a previous study.^23^

The model extended to 1, 3, and 5 years in order to estimate weight loss maintenance. Modelled patients could remain on active treatment for the duration of the modelled time horizon. Alternatively, patients could drop out of treatment or die from all causes. Dropout rates were estimated from the proportion of patients who dropped out of the respective clinical trials (Table 1). The therapy adherence rate was assumed to be constant for years 2 to 5. Mortality rates were estimated using BMI‐specific life tables from a previous analysis on the impact of obesity on mortality (Table S1).^39^ The model cycle length, or time between state transitions, was 1 month. All model inputs are listed in Table 1.

**Table 1:** Base case model inputs

	No Treatment	ILI	Phentermine	Phentermine/Topiramate	Liraglutide	Orlistat	Lorcaserin	Semaglutide
Monthly ΔBMI, year 1	0.0127^24^	−0.25^25^, ^26^, ^27^	−0.52^14^	−0.31^28^	−0.25^29^	−0.26^30^	−0.22^10^	−0.45^5^
Monthly ΔBMI, years 2 to 5	0.0127^24^	0.033^25^, ^26^, ^27^	0.14^14^	0.0029^28^	0.036^29^	0.04^30^	0.066^10^	0.0012^19^
Annual dropout rate (year 1), %	‐‐	2.8^25^, ^27^	35.0^14^	30.9^12^, ^28^	26.4^11^, ^29^, ^31^	31.4^30^	44.6^10^	18.0^5^
Annual dropout rate (years 2‐5), %	‐‐	2.2^25^, ^27^	44.0^14^	17.6^28^	19.0^29^	28.8^30^	27.4	3.25^5^, ^19^
Annual cost, $	‐‐	557^32^	623^18^, ^20^	1,647^20^	17,090^33^	1,234^34^	2,765^35^	8,273^36^
ΔQALY/ΔBMI	−0.0056^37^, ^38^							
Dropout ΔBMI, monthly	0.138^10^							

Abbreviations: BMI, body mass index; ILI, intensive lifestyle intervention; QALY, quality‐adjusted life year.

### 2.2 Strategies for weight management

Patients receiving no treatment experienced slight weight gain over time, based on published literature.^24^ This group served as a reference group composed of individuals not attempting any self‐directed weight loss. For patients in the treatment strategies, weight change was based on data from randomized, placebo‐controlled clinical trials (Table 2). In cases with more than one published clinical trial, the trial with the longest duration was selected. All pharmacotherapy clinical trials also included lifestyle modification counselling.

**Table 2:** Clinical trial data

Treatment	Baseline Weight (kg) /BMI, kg m^−2^	Sample Size	Weight After 1 y on Treatment, kg	Weight After 2 y on Treatment, kg	Source
ILI	100.5/35.89	2440	91.93	96.37	Look AHEAD trial^27^
Phentermine	98.4/35.6	269	81.08	85.90	Hendricks et al^14^
Phentermine/Topiramate	102.2/36.1	153	91.67	92.80	SEQUEL trial^28^
Liraglutide	97.6/34.8	93	89.3	91.5	Astrup et al^29^
Orlistat	100.7/36.2	153	91.84	93.05	Davidson et al^30^
Lorcaserin	100.4/36.2	1538	92.9	95.1	BLOOM trial^10^
Semaglutide	111.5/39.3	102	96.11	96.15	O'Neil et al^5^

Abbreviations: BMI, body mass index; ILI, intensive lifestyle intervention.

Most clinical trials contained data on weight loss for at least 2 years. For the model input, weight change was converted to rate of BMI change using average baseline weight and BMI values in the trial cohort (Table 1). Weight loss in the ILI strategy was based on data from the Look AHEAD study, which reported percent reduction from baseline weight over 8 years among type 2 diabetes patients receiving either ILI or diabetes support and education.^25^ For the 3‐year and 5‐year time horizons of the pharmacotherapy strategies, weight was assumed to increase linearly after the first year of pharmacotherapy. This assumption was based on the observed trends in the SCALE, BLOOM, and SEQUEL trials for liraglutide, lorcaserin, and phentermine/topiramate, respectively.^10^, ^28^, ^29^ For semaglutide, there were no clinical data past 1 year for a daily dose of 0.4 mg.^5^ However, the SUSTAIN‐6 trial followed type 2 diabetes patients on a weekly dose of 1.0 mg for 104 weeks.^19^ Change in weight after the first year from this study was used to estimate weight change on daily semaglutide after the first year in the model. For patients who dropped out of a weight loss strategy, the rate of weight regain was based on data from patients in the BLOOM trial who received lorcaserin in the first year and placebo in the second year.^10^ If a patient returned to baseline weight, the rate of weight gain was equivalent to patients on no treatment.

### 2.3 QOL adjustments and costs

QOL was dependent on weight change. A QOL constant of 0.0056 quality‐adjusted life years (QALYs) gained per BMI unit lost was used based on prior literature.^37^, ^38^

The model assumes a health care system cost perspective. Cost of no treatment was assumed to be zero. Costs of treatments were estimated from published literature (Table 1). The cost of semaglutide was estimated from the cost of once‐weekly 1.0 mg injections.^36^ For all pharmacotherapy arms, the cost for two physician visits ($178) was added to the first year, to account for the two visits expected for patients beginning weight loss medication.^20^ Costs of comorbidities and adverse events were not included. All costs from prior years were adjusted to 2019‐year dollars using the Consumer Price Index. Both costs and utilities were discounted at a rate of 3%.

### 2.4 Outcomes

Study endpoints included QALYs, total costs (US $2019), and incremental cost‐effectiveness ratios (ICERs). ICERs are calculated as the ratio of differences in costs and QALYs between a strategy and the next best alternative. A commonly used willingness‐to‐pay (WTP) threshold of $100 000/QALY determined cost‐effectiveness.^40^

### 2.5 Sensitivity analyses

To assess the impact of model input uncertainty on cost‐effectiveness results, one‐way sensitivity analyses and a probabilistic sensitivity analyses (PSAs) were performed. Deterministic one‐way sensitivity analyses were performed by varying one parameter at a time within prescribed bounds and recording the change in ICERs. All parameters were varied by +/−20% of the base case values. Probabilistic sensitivity analyses were performed by sampling all parameters simultaneously from probability distributions. The mean values for these distributions were the base case values for each parameter, and the standard deviations were 20% of the means. Gamma distributions were used for costs, and beta distributions were used for all other parameters. One thousand Monte Carlo samples were run per strategy with cohorts of 10 000 patients. The percentage of times each strategy was cost‐effective at various WTP thresholds was recorded.

## 3 RESULTS

### 3.1 Base case results

**Figure 1:**
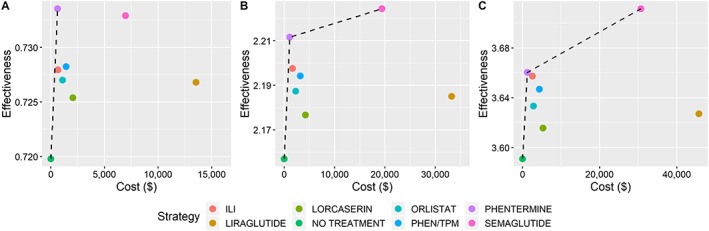
Base case results in cost‐effectiveness planes after (A) 1 year, (B) 3 years, and (C) 5 years. The dashed lines indicate the efficiency frontiers. PHEN/TPM, phentermine/topiramate

The results of the base case analysis are given in Table 3 and depicted as efficiency frontiers in Figure 1. An efficiency frontier plots cost and effectiveness for each strategy. Optimal strategies lie on the efficiency frontier (dashed line), while suboptimal (dominated) strategies lie below the frontier. For all time horizons, no treatment was the reference strategy (lowest cost and lowest effectiveness). Phentermine was the only strategy on the efficiency frontier after 1 year, dominating all other strategies. After years 3 and 5, semaglutide was also on the efficiency frontier.

**Table 3:** Base case results

	Cost, $	QALY	ICER, $/QALY	ICER Excluding Phentermine
Year 1				
No Treatment	0	0.720	‐‐	‐‐
Lorcaserin	2064.83	0.725	Dominated	Dominated
Liraglutide	13 533.25	0.727	Dominated	Dominated
Orlistat	1108.36	0.727	Dominated	Dominated
ILI	674.61	0.728	Dominated	82 733
Phentermine/Topiramate	1424.02	0.728	Dominated	Dominated
Semaglutide	6972.12	0.733	Dominated	1 267 325
Phentermine	636.02	0.734	46 258	
Year 3				
No Treatment	0	2.157	‐‐	‐‐
Lorcaserin	4229.10	2.177	Dominated	Dominated
Liraglutide	33 206.39	2.185	Dominated	Dominated
Orlistat	2284.81	2.187	Dominated	Dominated
Phentermine/Topiramate	3219.30	2.194	Dominated	Dominated
ILI	1673.68	2.198	Dominated	41 265
Phentermine	1100.83	2.212	20 157	
Semaglutide	19 375.75	2.224	1 437 340	661 326
Year 5				
No Treatment	0	3.591	‐‐	‐‐
Lorcaserin	5331.07	3.616	Dominated	Dominated
Liraglutide	45 744.97	3.627	Dominated	Dominated
Orlistat	2862.47	3.633	Dominated	Dominated
Phentermine/Topiramate	4402.22	3.657	Dominated	Dominated
ILI	2605.55	3.657	Dominated	39 219
Phentermine	1240.45	3.660	17 880	
Semaglutide	30 704.99	3.712	576 931	520 262

Abbreviations: ICER, incremental cost‐effectiveness ratio; ILI, intensive lifestyle intervention; QALY, quality‐adjusted life year.

Phentermine was the cost‐effective strategy for each time horizon, with ICERs of $46 258/QALY, $20 157/QALY, and $17 880/QALY after 1, 3, and 5 years, respectively. Weight loss in the first year was the greatest on phentermine, making it the most effective treatment in the first year. However, this weight loss was not sustained, and patients returned to baseline weight by year 5 (Figure S1).

By contrast, patients on semaglutide maintained significant weight loss throughout the 5‐year time horizon. As a result, semaglutide was the most effective strategy in later years, with total QALYs of 2.224 and 3.711 in years 3 and 5, respectively. However, semaglutide was not cost‐effective. ICERs were prohibitively high at $1 437 340/QALY and $576 931/QALY in years 3 and 5, respectively.

When excluding phentermine from the analysis, ILI became the cost‐effective strategy. In this scenario, the ICERs for ILI were $82 733/QALY, $41 265/QALY, and $39 219/QALY in years 1, 3, and 5, respectively. Semaglutide remains cost‐ineffective but with lower ICER values: $661 326/QALY and $520 262/QALY in years 3 and 5, respectively.

### 3.2 One‐way sensitivity results

**Figure 2:**
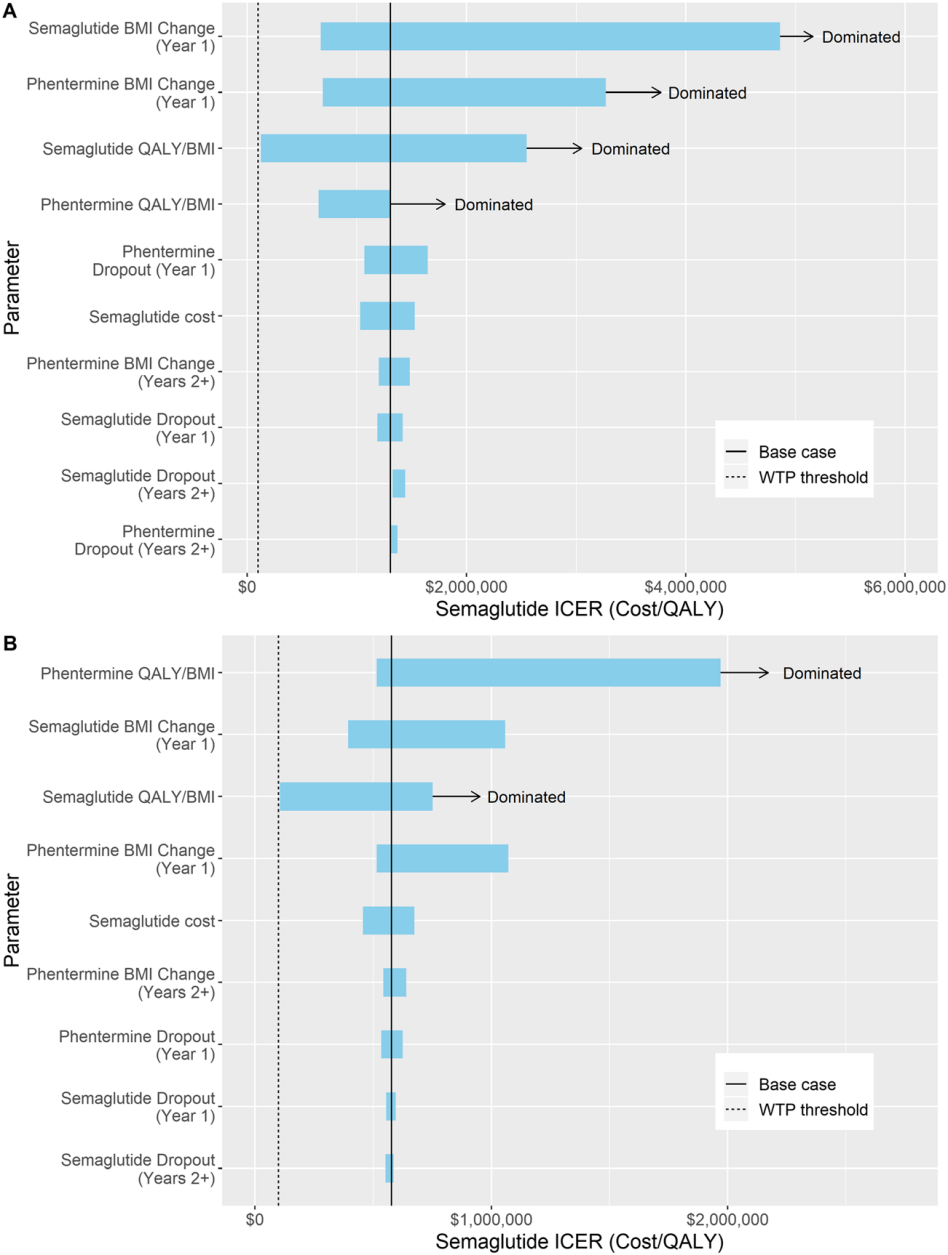
One‐way sensitivity analyses depicted as tornado diagrams. Range in semaglutide incremental cost‐effectiveness ratios (ICERs) when comparing phentermine vs semaglutide after (A) 3 years and (B) 5 years

Figure 2 depicts one‐way sensitivity analyses over 3‐ and 5‐year time horizons, corresponding to years with multiple strategies on the efficiency frontier. Each time horizon compares phentermine and semaglutide, the two strategies on the efficiency frontier.

For results over 3 years, changing the rate of BMI loss in year 1 and the QOL constant had substantial effects on resulting semaglutide ICERs. For each of these parameters, semaglutide was dominated under certain conditions. This was the case when the BMI lost in year 1 on phentermine was greater than 0.6 kg m^−2^, or the BMI lost in year 1 on semaglutide was less than 0.37 kg m^−2^. This was also the case when the QOL constant for phentermine was above 0.006 QALYs gained per BMI unit lost or the QOL constant for semaglutide was below 0.005 QALYs gained per BMI unit lost. Semaglutide approached the WTP threshold when its QOL constant was 0.017 QALYs gained per BMI unit lost, with an ICER of $127 062.

For results over 5 years, changing the rate of BMI loss in year 1 and the QOL constant also had the greatest effects on resulting semaglutide ICERs but not to the same extent as in the 3‐year time horizon. Semaglutide was dominated when the QOL constant for phentermine was above 0.009 QALYs gained per BMI unit lost or the QOL constant for semaglutide was below 0.004 QALYs gained per BMI unit lost. Semaglutide again approached the WTP threshold when its QOL constant was 0.017 QALYs gained per BMI unit lost, with an ICER of $106 873.

The results of these one‐way sensitivity analyses indicate that base case results were robust and phentermine remained the cost‐effective strategy under varying conditions. The semaglutide ICER did not fall below the WTP threshold in any test scenarios, although it became very close when the semaglutide QOL constant was much higher than the phentermine QOL constant.

### 3.3 PSA results

**Figure 3:**
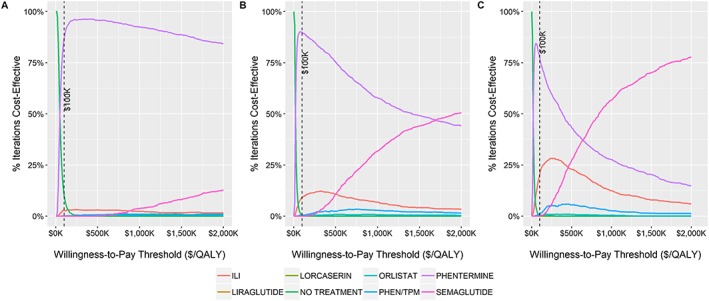
Probabilistic sensitivity analyses results depicted as acceptability curves after (A) 1 year, (B) 3 years, and (C) 5 years. The dashed line indicates the base case willingness‐to‐pay threshold. PHEN/TPM, phentermine/topiramate

**Figure 4:**
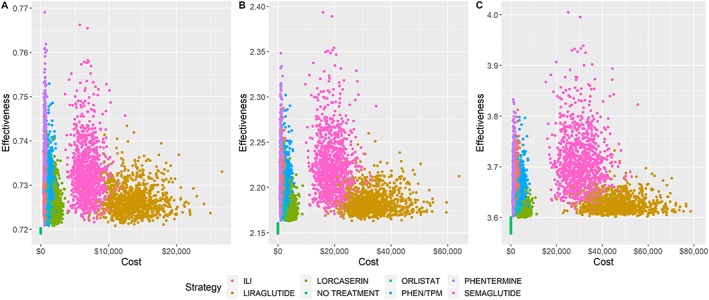
Probabilistic sensitivity analyses depicted in cost‐effectiveness planes after (A) 1 year, (B) 3 years, and (C) 5 years. PHEN/TPM, phentermine/topiramate

Probabilistic sensitivity analyses were performed on the model over the three time horizons. Acceptability curves are shown in Figure 3, and scatter plots of cost and effectiveness values are shown in Figure 4. The following results use a WTP threshold of $100 000/QALY. After 1 year, phentermine was the cost‐effective choice in 87.3% of runs, no treatment in 9.7% of runs, and ILI in 2.9% of runs. After 3 years, phentermine was the cost‐effective choice in 89.9% of runs and ILI in 9.2% of runs. After 5 years, phentermine was the cost‐effective choice in 77.4% of runs and ILI in 20.5% of runs.

## 4 DISCUSSION

The aim of this analysis was to determine cost‐effectiveness among six pharmacotherapies and ILI used to treat obesity. A simulation model was used as a platform to incorporate weight loss and QOL data from clinical trials and other published literature. The modelling results found that phentermine was the cost‐effective strategy over 1‐, 3‐, and 5‐year time horizons. Phentermine resulted in the most weight loss in the first year and was the least expensive pharmacotherapy. Since weight loss on phentermine was not as well sustained after the first year compared with other therapies, semaglutide became the most effective strategy over 3‐ and 5‐year time horizons. Weight loss on semaglutide was second only to phentermine in the first year and was maintained over the 5‐year time horizon. Despite its effectiveness, semaglutide did not achieve cost‐effectiveness because of its high cost.

Treatment decisions are highly specific to individual values and preferences, and phentermine may not be the best choice for everyone. Phentermine is not recommended for patients with a history of cardiovascular disease because of the medication's side effect profile.^15^ This cost‐effectiveness analysis aimed to provide data to inform treatment decision making from a particular framework. Other considerations important to an individual patient could include previous attempts to lose weight, existing comorbid conditions, use of other medications, treatment side effects, and patient preference.^7^, ^41^ For this reason as well as a lack of FDA approval for long‐term use, a separate scenario analysis was performed that excluded phentermine. In this scenario, ILI was the cost‐effective treatment. While it did not lead to the most weight loss, ILI was much less costly than the more effective alternatives: semaglutide and liraglutide.

When excluding patients with a history of cardiovascular events, a large electronic health record study found significantly greater weight loss among “off‐label” phentermine users (those who take the drug longer than the FDA‐approved 90 days) without any increase in risk for cardiovascular events over 3 years.^15^ In addition, given the widespread off‐label use of phentermine for over 20 years without any evidence of serious side effects, the Endocrine Society includes phentermine in their Clinical Practice Guideline, assuming educated patient preference, response to treatment, and no history of cardiovascular disease or increase in blood pressure or pulse while on treatment.^17^

Two previous cost‐effectiveness analyses have examined pharmacotherapies, along with other commercial weight loss programmes.^20^, ^21^ However, this study is the first cost‐effectiveness analysis to directly compare six pharmacotherapies. This is also the first analysis to incorporate data from the recent clinical trial on the efficacy and safety of daily semaglutide for the treatment of obesity. In addition, other similar analyses did not include phentermine. A variety of treatments were included in the analysis in order to reflect decision making in clinical practice, primarily for patients who have unsuccessfully attempted weight loss with lifestyle intervention.

There were limitations to this analysis that should be acknowledged. This model did not directly incorporate adverse effects or side effects of treatment. Instead, QOL was dependent solely on weight loss. This was due to insufficient data regarding quality of life and the side effects of each drug. Treatment adherence was incorporated, however, which directly impacts therapy effectiveness and is largely dependent on adverse events and side effects. In addition, since most clinical trials included data for only 1 or 2 years, changes in weight were extrapolated past the first year for the extended time analyses with the assumption that weight linearly increased. This was based on the plateau or slight increase seen in many clinical trials after about 40 weeks. It was possible for patients to remain on treatment for all 5 years, which is uncommon and, in some cases, not recommended for some pharmacotherapies, particularly phentermine. In addition, many patients take medications intermittently, which was not considered in this modelling analysis. However, longer term continuous treatment regimens are becoming more common in accordance with the growing understanding of obesity as a chronic disease.^15^ Extensive sensitivity analyses were performed to evaluate the uncertainty resulting from model assumptions and indicated that the results were consistent despite changes in the parameters.

In summary, this modelling analysis found that phentermine is the cost‐effective pharmacotherapy currently on the market. This highlights the influence of drug cost and the need for further research into chronic therapy for patients with obesity. Longer‐term clinical trials that fully capture QOL, weight loss, comorbidities, and adverse events are needed to confirm these findings.

## CONFLICT OF INTEREST STATEMENT

Dr Hur received consulting fees from Novo Nordisk outside the submitted work. Dr Corey received consulting fees from Bristol Myers Squibb, Novo Nordisk, and Gilead outside the submitted work and grant funding from Bristol Myers Squibb and Boehringer‐Ingelheim. Dr Kaplan is a consultant to Novo Nordisk. Novo Nordisk manufactures liraglutide and semaglutide.

## Supporting information

Data S1. Supporting information
